# MicroRNA Expression in Selected Carcinomas of the Gastrointestinal Tract

**DOI:** 10.4061/2011/124608

**Published:** 2011-02-13

**Authors:** Nicole C. Panarelli, Rhonda K. Yantiss

**Affiliations:** Department of Pathology and Laboratory Medicine, Weill Cornell Medical College, 1300 York Avenue, New York, NY 10065, USA

## Abstract

MicroRNAs (miRNAs) comprise a recently discovered class of small, 18–25 nucleotide, noncoding RNA sequences that regulate gene expression at the posttranscriptional level by binding to and inhibiting the translation of target messenger RNAs (mRNAs). Characteristic patterns of miRNA expression have been described in several malignancies of the gastrointestinal tract, and numerous investigators have demonstrated interactions between specific miRNA species and target oncogenes or tumor-suppressor genes. It is clear that miRNAs play an important role in regulating expression of a number of genes involved in gastrointestinal carcinogenesis, and, thus, these molecules may represent either diagnostic markers of, or therapeutic targets for, some types of malignancy. This paper summarizes the literature regarding miRNA expression in carcinomas of the colon, pancreas, and liver and discusses some of the mechanisms by which these molecules participate in gastrointestinal oncogenesis.

## 1. Introduction

MicroRNAs are small, 18–25 nucleotide, noncoding RNA sequences that regulate gene expression at the posttranscriptional level by binding to and inhibiting translation of target messenger RNAs (mRNAs). Over 1,000 human miRNAs have been identified to date, and many have tissue-specific expression profiles. Several studies have shown that miRNAs demonstrate characteristic patterns of expression in cancers. Some species show overexpression in cancers relative to nonneoplastic tissues, whereas others display decreased expression, similar to oncogenes and tumor suppressor genes, respectively [1–9]. Emerging data indicate that virtually every type of human malignancy displays dysregulated miRNA expression, and, in fact, potential applications of miRNA expression profiling to the diagnosis, prognosis, and treatment of gastrointestinal cancers have been the subject of extensive recent investigation. Some investigators have suggested that panels of miRNAs may be used for diagnostic purposes among patients with suspected gastrointestinal malignancies, and a subset of these represent prognostically important markers or even potential therapeutic targets. The purpose of this paper is to provide the readership with a comprehensive overview of published data regarding miRNA expression in gastrointestinal malignances with emphasis on colorectal, pancreatic, and hepatocellular carcinomas. 

## 2. Overview

MicroRNAs were first recognized as regulatory agents of gene expression in 1993 when they were discovered in *Caenorhabditis elegans *[10]. Each miRNA molecule can potentially bind to either the 3′- or 5′-untranslated region (UTR) of hundreds of mRNAs by sequence complementarity. Binding of miRNA to mRNA suppresses expression by either inducing mRNA degradation or inhibiting translational machinery. Primary miRNA transcripts within the nucleus are processed by the nuclear RNAse III complex, Drosha, to become miRNA precursors termed “pre-miRNAs”. Pre-miRNAs are exported to the cytoplasm where the endoribonuclease, Dicer, processes them into mature miRNAs. These mature molecules are subsequently integrated into the RNA-induced silencing complex (RISC), the functional unit of which inhibits mRNA translation [11, 12]. 

A growing body of evidence indicates that a subset of miRNAs are functionally important to the development of human cancers. Many investigators have identified tumor-specific miRNA signatures that accurately distinguish malignancies from benign tissues in multiple different sites, suggesting that some miRNAs are oncogenic, and their potency depends on other gene mutations that are present in the tumor. Manipulation of miRNAs in cancer cell lines directly affects cell proliferation and apoptosis, and many researchers have demonstrated links between miRNA dysregulation and cell signaling pathway abnormalities [9, 13–15]. Thus, miRNAs comprise a recently described class of molecules that contributes to cancer formation through interactions with mRNAs derived from oncogenes and tumor suppressor genes. 

## 3. MicroRNAs in Colorectal Cancer

MicroRNA expression in colon cancer and nonneoplastic colonic tissues has been extensively studied (Table 1). Cummins et al. performed serial analysis of gene expression (miRAGE) in colorectal cancer cell lines and identified 133 miRAGE tags that corresponded to previously unrecognized miRNAs. They also detected differential expression of 52 miRAGE tags in colon cancer cells relative to normal colonic epithelium. These results provided evidence that the number of miRNAs in the human genome is likely much larger than had been previously predicted and that their expression is frequently dysregulated in colorectal cancer [25]. Subsequent studies provided data indicating that miRNA dysregulation is important to colon cancer development. Bandres et al. studied expression of 156 miRNAs in colon cancer cell lines as well as paired tumoral and nontumoral tissues. They identified a subset of 13 differentially expressed species [16]. Wang et al. used miRNA microarrays to identify 12 miRNAs that were upregulated in colon cancer and 2 that were downregulated compared to nonneoplastic colonic tissues [17].

The expression levels of several miRNA species have been associated with clinicopathologic features and prognosis in colon cancer. MicroRNA-31 was first identified by Bandres et al. as one of the most substantially dysregulated miRNAs in colon cancer cell lines and resected colon cancers. The authors of that study found that miR-31 expression was significantly higher in stage IV tumors compared to stage II carcinomas [16]. Wang et al. later demonstrated an association between miR-31 upregulation and advanced TNM stage as well as deeper invasion of the primary tumor [26]. Slaby et al. failed to identify any correlation between miR-31 expression and tumor stage in their analysis of 29 colon carcinomas, but they did note that miR-31 levels were significantly higher in high-grade carcinomas, compared to low-grade tumors [19]. MicroRNA-21, a species with antiapoptotic properties, is dysregulated in many human cancers including tumors of the head and neck, lung, breast, prostate, brain, thyroid, pancreas, stomach, colon, and esophagus [9, 27–32]. Its high expression has been associated with regional lymph node and distant metastases in colorectal cancer patients [19]. Schetter et al. analyzed 197 colonic adenocarcinomas using microarray assays and qRT-PCR and found that high miR-21 levels predicted poor survival prognosis and higher TNM stage [21]. This same group later demonstrated a relationship between high miR-21 expression and increased levels of IL-6, a proinflammatory cytokine and lower levels of IL-12a in colonic adenocarcinomas. They postulated that IL-6 drives miR-21 expression whereas IL-12a is a negatively regulated target of miR-21 [21]. Presumably, IL-12a activity is important for host resistance to malignancy. Thus, its downregulation by miR-21 may account for some of the negative impact of miR-21 on prognosis among patients with colorectal cancer. Finally, miR-145 is normally expressed in colonic epithelium, but it shows decreased expression in colon cancer [23]. Decreased miR-145 is more commonly observed in tumors of the proximal colon and those of large size (>50 mm) [22]. Akao et al. found decreased expression of miR-143 and miR-145 in adenomas and carcinomas, but they did not find any correlation between their expression and any other clinical prognostic factors, indicating that these miRNAs may primarily contribute to initiation, but not progression, of colonic tumorigenesis. Notably, synthetic miR-143 has a suppressive effect on growth of xenografted tumors comprised of human colon cancer cells [33].

 Expression of a number of other miRNA species has been reported to correlate with clinicopathologic features and prognosis among patients with colon cancer. Schepeler et al. found that stage II colon cancers with high miR-320 or miR-498 expression showed significant differences with respect to progression-free survival compared to tumors with low expression of these species [18]. High expression of miR-200c has also been reported to predict shorter survival and is associated with frequent p53 mutations [20]. Diaz et al. studied 110 patients with colon cancer and reported that downregulation of miR-106a predicted shorter disease-free survival [34]. Sarver et al. identified 6 miRNAs (HS-29, miR-135b, miR-32, miR-33, miR-542-5p, and miR-96) that were more highly expressed in stage IV microsatellite stable cancers relative to stage II tumors [22]. Huang et al. studied nonneoplastic colonic mucosa adjacent to colon cancers in three patients with lymph node metastases and three patients with node negative disease. They found 6-fold higher expression of miR-137 in lymph node positive tumors compared to node-negative cases [35]. Finally, Yantiss et al. studied miRNA expression in colorectal cancers obtained from 24 patients <40 years of age and 45 patients >40 years old. They found significantly increased expression of miR-21, miR-20a, miR-145, miR-181b, and miR-203 in tumors from young patients compared to older adults [36].

Data from several studies have documented interactions between specific miRNA species, oncogenes, and tumor suppressor genes relevant to colonic carcinogenesis. Chen et al. observed an inverse correlation between miR-143 and *KRAS* expression in 13 colonic adenocarcinomas. They also used semiquantitative RT-PCR to show that *KRAS* transcript levels were decreased in cell lines transfected with pre-miR-143 whereas addition of anti-miR-143 oligonucleotides increased *KRAS* transcript levels. These findings suggest that miR-143 downregulation in colon carcinoma promotes cancer cell growth by disinhibiting *KRAS* translation [37]. 

MicroRNAs also impact the Wnt signaling pathway. The adenomatous polyposis coli (*APC*) gene normally functions as a tumor suppressor by regulating Wnt signaling. In the absence of functional *APC*, *β*-catenin accumulates in the cytoplasm and is transported to the nucleus, where it facilitates transcription of genes involved in proliferation, such as *cyclin D1*. Germline *APC* mutations are responsible for familial adenomatous polyposis syndrome, and biallelic inactivation of *APC* occurs in the majority of sporadic colonic adenocarcinomas [38–41]. Transduction of colon cancer cell lines with miR-135a and miR-135b results in diminished *APC* expression and accumulation of *β*-catenin. Colon cancers with high levels of miR-135a and miR-135b show lower *APC* expression. In this situation, qRT-PCR data demonstrate reduced *APC* mRNA, suggesting that miR-135a and miR-135b regulate the Wnt signaling pathway by promoting mRNA decay [42]. 

MicroRNA-34, a species that is lost in multiple types of cancer including those of the colon and pancreas, is inducible by p53, and its overexpression is associated with p53 effects including cell cycle arrest and apoptosis [43]. Guo et al. found that restoration of miR-126, a species that shows low-to-absent expression in colon cancer, impedes cancer cell growth by targeting the p85B subunit of phosphatidylinositol-3-kinase (PI3K). Phosphatidylinositol-3-kinase activates AKT, a protein kinase involved in the PI3K/AKT/mTOR pathway, which triggers a variety of downstream responses related to cell growth, proliferation, and motility. Presumably, loss of miR-126 removes a critical checkpoint in the PI3K/AKT/mTOR pathway and facilitates tumor growth [24]. Continued research will likely uncover additional regulatory roles for miRNAs in colorectal carcinogenesis and other neoplasms throughout the gastrointestinal tract.

Most colorectal cancers are characterized by aneuploidy, allelic imbalance, and mutations in *KRAS*, *TP53*, and *APC* although approximately 15% of sporadic colonic adenocarcinomas develop *via* microsatellite instability (MSI) and have defective DNA mismatch repair mechanisms. Such tumors are often located in the proximal colon, show high-grade histology, and contain infiltrating lymphocytes. They are generally diploid and have a better prognosis than non-MSI tumors, but they are probably less responsive to conventional 5-flourouracil-based chemotherapy [44, 45]. Patients with germline mutations in one of the mismatch repair genes (*MLH1*, *MSH2*, *MSH6*, and *PMS2*) are at risk for acquired mutations of the second allele and development of heritable microsatellite unstable carcinomas of the gastrointestinal, genitourinary, and gynecologic tracts, termed Lynch syndrome. Sporadic colonic carcinomas with MSI may also occur, but in this situation, tumorigenesis results from biallelic epigenetic methylation and silencing of *MLH1*. Several authors have studied differences in miRNA expression patterns between microsatellite stable (MSS) colon cancers and those with MSI-H (Table 2). Lanza et al. used microarray data to profile 23 MSS and 16 sporadic MSI-H colon cancers. They identified 72 mRNAs and 14 miRNAs that were differentially expressed between these two tumor types and used their combined expression to distinguish between MSS and MSI-H cancers [14]. Sarver et al. studied expression of 735 miRNAs in 12 MSI-H and 68 MSS colon tumors and found that miR-552, miR-592, miR-181c, and miR-196b were significantly increased in MSS tumors relative to MSI-H tumors, whereas miR-625 and miR-31 levels were higher in MSI-H tumors [22]. Schepeler et al. compared miRNA expression in 37 MSS relative to 12 MSI-H cancers and identified a 4-miRNA signature (miR-142-3p, miR-212, miR-151, and miR-144) that predicted MSI with 81% specificity and 92% sensitivity [18]. These data indicate that the type of genetic instability in colorectal cancer is reflected at the miRNA level.

5-Flourouracil (5-FU) has been a mainstay of colorectal cancer therapy for the past several decades [46]. Some patients have a suboptimal response to therapy for unclear reasons, so identification of molecular markers that predict the likelihood of a therapeutic response is important. *In vitro* studies evaluating miRNA expression in colon cancer cell lines treated with 5-FU have generated promising data regarding the potential use of miRNAs as markers of chemosensitivity. Borralho et al. showed that stable expression of miR-143, a species known to be downregulated in colon cancer, was associated with increased death in cell lines after exposure to 5-FU [23, 47]. Others have applied this concept to colon cancer resection specimens. Nakajima et al. used qRT-PCR to study miRNA expression in residual or recurrent colon cancers from 46 patients who were treated with oral 5-FU alone or in combination with cisplatin. Twenty-seven patients who experienced complete disease remission showed a partial response or maintained stable disease after treatment had significantly lower levels of let-7g and miR-181b, compared to 19 patients who suffered disease progression [48]. Schetter et al. analyzed associations between miR-21 expression and therapeutic outcomes in 56 stage II or stage III colorectal cancer patients treated with 5-FU. High miR-21 expression was associated with worse overall survival, lending preliminary support to the notion that miR-21 overexpression predicts a poor response to therapy [21]. Boni et al. investigated associations between polymorphisms in miRNA-containing genomic regions and genes related to miRNA biogenesis and clinical outcome in patients with metastatic colon cancer who were treated with 5-FU and irinotecan. Single-nucleotide polymorphisms in the miR-26-a-1 gene and 5′UTR of pre-miR-100 correlated with better overall survival and disease control, respectively, and both were associated with a prolonged interval to progression [49]. The mechanisms by which miRNAs modulate efficacy of therapy are not understood, but these early data support the hypothesis that changes in miRNA expression levels and in the miRNA genome impact tumor response to therapy. 

## 4. MicroRNAs in Pancreatic Neoplasia

Aberrant miRNA expression has been described in pancreatic ductal adenocarcinoma and benign pancreatobiliary disease (Table 3). Early studies exploited differences in miRNA expression patterns to distinguish between benign and malignant pancreatic diseases. Bloomston et al. examined 65 pancreatic ductal adenocarcinomas and benign adjacent pancreatic tissue, as well as 42 cases of chronic pancreatitis for miRNA expression. They found 21 miRNAs to be dysregulated in cancer compared to benign tissues and noted that a panel of 11 miRNAs (miR-148a, miR-148b, miR-155, miR-181a, miR-181b, miR-181b-1, miR-181c, miR-181d, miR-21, miR-221, and miR-375) distinguished pancreatic ductal adenocarcinoma from chronic pancreatitis and normal pancreatic tissue [49]. Lee et al. analyzed 28 pancreatic ductal adenocarcinomas and 21 nonneoplastic pancreatic tissues and found miR-155, miR-181b, miR-181c, miR-21, and miR-221 to be among the top 20 of 100 miRNAs overexpressed in pancreatic cancer [31]. Szafranska et al. identified 26 dysregulated miRNAs in pancreatic adenocarcinoma using resection specimens and pancreatic cancer cell lines. They reported that miR-196a upregulation combined with miR-217 downregulation reliably distinguished pancreatic cancer from benign pancreas and chronic pancreatitis [50]. In a later study, this signature correctly identified malignancy in 9/10 fine needle aspiration biopsies of pancreatic cancer [51]. 

The roles of dysregulated miRNAs in neoplastic pancreatic lesions have also been examined. du Rieu et al. used qRT-PCR to study miRNA expression in pancreatic intraepithelial neoplasia (PanIN) samples from human and mouse pancreas and found that levels of miR-21, miR-221, miR-22, and let-7a increased in the progression of PanIN to carcinoma [52]. Dilhoff et al. found that strong miR-21 expression by *in situ *hybridization was associated with decreased survival among patients with lymph node-negative pancreatic carcinoma [53]. Ikenaga et al. used qRT-PCR to evaluate miR-203 levels in resection specimens from patients with pancreatic ductal adenocarcinoma (*n* = 113), chronic pancreatitis (*n* = 20), and samples of nondiseased pancreas (*n* = 8) and found higher expression of miR-203 in pancreatic cancer. Results of a multivariate analysis showed miR-203 expression to be an independent predictor of poor prognosis in patients who had undergone complete tumor resection [54]. Wang et al. investigated expression of miR-21, -210, -155, and 196a by qRT-PCR in the sera of 49 patients with pancreatic adenocarcinoma and 36 healthy controls and found higher expression of all four markers in sera of pancreatic cancer patients [55]. In a later study, Kong et al. analyzed serum levels of miR-196a, miR-21, and miR-155 in 35 patients with pancreatic adenocarcinoma, 15 patients with chronic pancreatitis, and 15 healthy controls. They found that higher miR-21 expression distinguished cancer patients from those with chronic pancreatitis and healthy subjects, whereas miR-155 and miR-196a discriminated between patients with chronic pancreatitis and healthy controls. They also noted that serum miR-196a levels were significantly higher in patients with unresectable cancer than in those amenable to surgery and that higher miR-196a levels predicted shorter survival in pancreatic cancer patients [56]. Others have shown increased miR-196a expression to be associated with a 2-year survival of 17%, compared to 64% among tumors with low expression of this marker [30].

The relative tissue-specificity of miRNAs makes them attractive targets for molecular therapy among patients with pancreatic cancer. Park et al. analyzed the effects of miR-21 and miR-221 antisense oligonucleotides on pancreatic cancer cell lines and found that treated cells showed increased apoptosis and cell cycle arrest compared to cells treated with control oligonucleotides. MicroRNA-21 targets two tumor suppressor molecules, PTEN and RECK, both of which were found to be increased in extracts from cell lines treated with antisense oligonucleotides to miR-21. Similarly, p27, the target of miR-221, increased when this molecule was inhibited. Park et al. also found that cells pretreated with antisense sequences against miR-21 and miR-221 showed decreased viability by colorimetric analysis following gemcitabine treatment compared to those treated with control oligonucleotides [57].

Few studies have investigated miRNA expression in pancreatic endocrine and acinar tumors. Roldo et al. performed microarray and northern blot analyses of 40 endocrine tumors, 4 acinar cell carcinomas, and 12 samples of nonneoplastic pancreas. They found that stable expression of miR-103 and miR-107, in combination with a lack of miR-155 expression, discriminated all tumor samples from nonneoplastic tissues. They also identified 10 miRNAs that distinguished endocrine from acinar tumors and 28 miRNA species that were aberrantly increased in both tumor types [29]. These preliminary data suggest that altered miRNA expression occurs in endocrine and acinar neoplasms of the pancreas. 

## 5. MicroRNAs in Liver Disease

Early studies evaluating miRNA expression in hepatocellular carcinoma identified approximately 69 dysregulated species (Table 4) [58–63]. Many of those miRNAs, including miR-122, miR-221, and miR-222, were later recognized as carcinogenic catalysts and prognostic markers in hepatocellular carcinoma. MicroRNA-122 is the most abundant miRNA in hepatic parenchyma and is relatively specific for hepatocyte differentiation, showing rare expression outside of liver [64–66]. Downregulation of miR-122 is frequently observed in hepatocellular carcinoma, and hepatocellular carcinoma cell lines treated with miR-122 oligonucleotides show increased apoptosis and decreased viability [58, 59, 67–72]. Coulouarn et al. correlated miR-122 tissue levels with the clinicopathologic features of 64 hepatocellular carcinomas and found that low expression of this marker predicted shorter survival, high-grade histology, and large tumor size. They also noted that loss of miR-122 was associated with higher expression of genes involved in cell motility, angiogenesis, hypoxia, and epithelial-mesenchymal transition [73]. Tsai et al. reported lower levels of miR-122 in hepatocellular carcinomas with intrahepatic metastases compared to solitary tumors [74]. 

 The roles of microRNA species in hepatocellular carcinogenesis and their molecular targets are under current investigation. Wong et al. found that high miR-222 levels correlated with advanced tumor stage and shorter overall survival in patients with hepatocellular carcinoma independent of stage. They also noted PI3K/AKT/mTOR pathway inhibition occurred in hepatocellular carcinoma cell lines transfected with anti-miR-222 [62]. MicroRNA-221, another frequently dysregulated species in hepatocellular carcinoma, participates in the modulation of key molecules related to hepatocarcinogenesis. Pineau et al. found that expression levels of the cyclin-dependent kinase inhibitor, p27, and the PI3K/AKT/mTOR pathway regulator, DDIT4, were decreased in liver cancer cell lines that overexpressed miR-221 [61]. Gramantieri et al. showed an inverse correlation between miR-221 upregulation and levels of the proapoptotic protein, Bmf, in hepatocellular carcinoma samples [76]. Fornari et al. showed increased CDKN1C/p57 and CDKN1B/p27 protein levels by western blot analysis in hepatocellular carcinoma cell lines transfected with anti-miR-221 compared to controls. Conversely, cell lines treated with miR-221 showed decreased CDKN1C/p57 and CDKN1B/p27 protein levels [77]. Finally, Mneg et al. reported that inhibition of miR-21 in hepatocellular carcinoma cell lines increased expression of PTEN and decreased tumor cell proliferation, suggesting that increased miR-21 levels promote carcinogenesis [78]. Other identified, but less-well-studied, modulators of apoptosis in hepatocellular carcinoma include miR-29, miR-15b, miR-152, miR-101, and the miR-106b-25 cluster [75, 79–82].

Ji et al. evaluated a cohort of 214 patients with hepatocellular carcinoma and found that tumors with reduced miR-26 expression had a favorable response to adjuvant therapy with interferon alpha, whereas those with high miR-26 did not respond to therapy, suggesting that miR-26 may be used to select patients who may benefit from interferon alpha treatment [83]. Connelly et al. reported that miR-21 and the miR-17-92 polycistron are consistently upregulated in human and animal hepatocellular carcinoma cell lines and that their inhibition by antisense oligonucleotides causes reduced tumor cell proliferation [84]. 

Recent studies have established a role for miRNAs in regulation of hepatitis C virus (HCV) infection and offer promise for new treatment modalitites. The most frequently implicated miRNA in HCV modulation is the liver-specific species, miR-122. Jopling et al. first described a physical interaction between miR-122 and the HCV genome by showing that miR-122 binds to the 5′ UTR of viral RNA and stimulates viral replication [85]. Henke et al. showed that miR-122 drives HCV translation by enhancing the association between a small ribosomal subunit and HCV RNA [86]. Both mechanisms of HCV potentiation were later validated by Jangra et al. who demonstrated that viruses with mutations in miR-122 binding sites failed to replicate [87]. Young et al. reported decreased viral replication in liver cells treated with inhibitors of miR-122 and suggested that these small molecules may represent a new target for HCV therapy, which has already been successfully tested in HCV-infected chimpanzees [72, 88]. 

The potential impact of miRNA analysis on patient selection for specific therapies was underscored by Sarasin-Filipowicz et al. These authors assessed miR-122 levels by qRT-PCR in pre- and posttreatment liver biopsies from 42 patients with HCV. They found that patients with decreased miR-122 levels in pretreatment liver biopsies showed a poor response to interferon therapy [89]. Other miRNA species such as miR-24, miR-149, miR-638, and miR-1181 have also been implicated in HCV-related liver disease and may facilitate viral entry, replication, and propagation [90]. 

MicroRNA dysregulation also occurs in association with hepatitis B virus (HBV) infection and may provide clues to the pathogenesis of HBV-related disease in infected patients. Yang et al. found that miR-602 expression increased with progression of HBV-related hepatitis to cirrhosis and hepatocellular carcinoma and noted that the tumor suppressor gene *RASSF1A* was inhibited in cell lines that highly expressed miR-602 [91]. Ura et al. studied 12 patients with HBV-related hepatocellular carcinoma and 14 with HCV-related hepatocellular carcinoma. They identified 19 differentially expressed miRNAs between patients with HBV and HCV infection. Microarray analysis also identified separate target genes for HBV- and HCV-related cancers. MicroRNAs important to HBV-related carcinoma regulate genes involved in cell death, DNA damage, recombination, and signal transduction whereas those important to HCV-related carcinoma were related to immune response, antigen presentation, cell cycle, and proteasome and lipid metabolism [92]. These findings provide insight into the differences between HBV- and HCV-infection and disease progression and may help identify potential therapeutic target molecules in the future. 

## 6. MicroRNAs and *In Vitro* Cancer Models

Several strategies utilizing miRNAs as *in vivo *therapeutic targets are currently under development. Use of antisense oligonucleotides has been most extensively studied *in vitro*, and was recently shown to be an effective suppressor of miRNA expression *in vivo*. Krutzfeldt et al. engineered synthetic RNA analogues, termed “antagomiRs,” to miR-16, miR-122, miR-192, and miR-194. These compounds were administered to mice intravenously and corresponding miRNA levels were measured by northern blot assay 24 hours after injection. These authors reported a marked reduction in target miRNA levels in various tissues including liver, lung, kidney, heart, intestine, fat, skin, bone marrow, muscle, ovaries, and adrenal glands [93]. Locked nucleic acid (LNA) constructs represent another promising approach to suppressing miRNA expression. These molecules are nucleic acid analogues that are “locked” by a methylene bridge connecting the 2'O and 4'C atoms. This structural modification enables LNA oligonucleotides to bind complementary nucleotide sequences with high affinity and excellent mismatch discrimination. Elmen et al. administered an LNA-antimiR to African green monkeys in order to study its effect on plasma cholesterol levels and miR-122 levels in liver tissue by northern blot analysis. They observed a dose-dependent decrease in total plasma cholesterol and depletion of mature miR-122 in liver biopsies from these monkeys [94]. Finally, some investigators have employed adenovirus vectors to increase expression of tumor-suppressor miRNAs. Kota et al. showed that the adenovirus vector-mediated introduction of miR-26, a species downregulated in hepatocellular carcinoma, into mice with liver cancer caused cancer cell apotosis and tumor regression. This therapy had no adverse effect on benign hepatocytes, underscoring the potential applications of this approach [95]. Future *in vivo* progress in this field will depend upon increased understanding of miRNA function in mammals, improved chemical design of antimiRs and synthetic miRNAs, and development of more efficient methods for delivery of these molecules to target tissues. 

## 7. Conclusion

MicroRNAs represent an important class of molecules with profound diagnostic and therapeutic implications. Emerging evidence suggests that they may be useful diagnostic adjuncts that aid identification of tumors of unknown origin or even ascertain the presence of malignancy in scant biopsy specimens or sera of patients with suspected cancer. Specific miRNA expression profiles clearly correlate with prognosis, so it is highly likely that miRNA analysis will play an important role in determining the management of patients in the future. Preliminary studies utilizing antisense oligonucleotides against cancer-specific miRNAs have shown that some tumors respond to therapy while minimally damaging healthy tissues. These findings suggest that targeted therapies against selected miRNAs represent a new treatment modality for patients with gastrointestinal malignancies. Advances in this field have improved our understanding of the heterogeneity of human malignancies and will contribute to the growing trend toward individualized management strategies for cancer patients. 

## Figures and Tables

**Table 1:** Summary of microRNA expression in colorectal cancer.

Study	Differentially expressed miRNAs (no./total)	Most significantly overexpressed miRNAs in colorectal cancer	Most significantly underexpressed miRNAs in colorectal cancer
Bandres et al. [16]	13/156	miR-31, miR-96, miR-133b, miR-135b, miR-145, miR-183	
Wang et al. [17]	14/723	miR-106b, miR-135b, miR-18a, miR-18b, miR-196b, miR-19a, miR-224, miR-335, miR-424, miR-20a*, miR-301b, miR-734a	miR-378, miR-378*
Schepeler et al. [18]	60/315	miR-20a, miR-510, miR-92, miR-513	miR-145, miR-455, miR-484, miR-101
Slaby et al. [19]	4/4	miR-31, miR-21	miR-145, miR-143
Xi et al. [20]	4/10	miR-15b, miR-181b, miR-191, miR-200c	
Schetter et al. [21]	37/389	miR-20a, miR-21, miR-106a, miR-181b, miR-203	
Sarver et al. [22]	39/735	miR-135b, miR-96, miR-182, miR-182*, miR-183	miR-1, miR-133a, miR-30a-3p, miR-30a-5p, miR-20b, miR-363
Michael et al. [23]	2/28		miR-143, miR-145
Guo et al. [24]	45/262	miR-93, miR-92, miR-520h, miR-508, miR-505, miR-449, miR-429, miR-384, miR-373, miR-34c, miR-326, miR-25, miR-224, miR-210, miR-200a, miR-19b, miR-19a, miR-18a, miR-183, miR-182, miR-181b, miR-181a, miR-181c, miR-17-5p, miR-148a, miR-141, miR-130b, miR-128a, miR-106b, miR-106a, let-7d	miR-96, miR-485-5p, miR-422b, miR-342, miR-214, miR-199a, miR-195, miR-150, miR-145, miR-143, miR-133a, miR-126, miR-125b, miR-100

**Table 2:** MicroRNA expression in microsatellite stable *versus* microsatellite unstable colorectal cancer.

Study	MSS colorectal cancers		MSI-H colorectal cancers	
	Overexpressed	Underexpressed	Overexpressed	Underexpressed
Schepeler et al. [18]	miR-20a, miR-510, miR-92, miR-513	miR-142-3p, miR-212, miR-146b, miR-217	miR-492, miR-20a, miR-432*, miR-21	miR-145, miR-455, miR-484, miR-101
Sarver et al. [22]		miR-552, miR-592, miR-181c, miR-196b	miR-625, miR-31	
Lanza et al. [14]	miR-215, miR-192, miR-191, miR-203, miR-32, miR-17, miR-25, miR-106a, miR-92-1, miR-92-2, miR-93-1, miR-20		miR-223, miR-155	

**Table 3:** Summary of microRNA expression in pancreatic ductal adenocarcinoma.

Study	Differentially expressed miRNAs (no./total)	Most significantly overexpressed miRNAs compared with normal controls and/or chronic pancreatitis	Most significantly underexpressed miRNAs compared with normal controls and/or chronic pancreatitis
Bloomston et al. [30]	46/326	miR-221, miR-181a, miR-155, miR-210, miR-213, miR-181b, miR-222, miR-181b-2, miR-21, miR-181b-1, miR-181c, miR-220, miR-181d, miR-223, miR-100-1/2, miR-125a, miR-143, miR-10a, miR-146, miR-99, miR-100, miR-199a-1, miR-10b, miR-199a-2, miR-107, miR-103-2, miR-125b-1, miR-205, miR-23b, miR-23a, miR-96, miR-34, miR-497, miR-203, miR-453, miR-92, miR-93, miR-21	miR-148a, miR-148b, miR-375, miR-494, miR-483, miR-339, miR-218-2, miR-409-3p

Lee et al. [31]	100/222	miR-221, miR-424, miR-301, miR-100, miR-376a, miR-125b-1, miR-21, miR-16-1, miR-181a, miR-181c, miR-92-1, miR-15b, miR-155, let-7f-1, miR-212, miR-107, miR-24-1, miR-24-2, let-7d	miR-345, miR-142-p, miR-139

Szafranska et al. [50]	26/377	miR-205, miR-143, miR-145, miR-146a, miR-148a, miR-196b, miR-93, miR-31, miR-210, miR-196a, miR-18a, miR-203, miR-150, miR-155, miR-221, miR-222, miR-223, miR-224	miR-29c, miR-216, miR-217, miR-375, miR-148a, miR-96, miR-148b, miR-141, miR-130b

du Rieu et al. [52]	7/7	miR-21, miR-221, miR-222, miR-200, miR-205, miR-29c	let-7a

**Table 4:** Summary of microRNA expression in hepatocellular carcinoma.

Study	Differentially expressed miRNAs (no./total)	Most significantly overexpressed miRNAs compared with nonneoplastic liver (no disease or chronic viral hepatitis)	Most significantly underexpressed miRNAs compared with nonneoplastic liver (no disease or chronic viral hepatitis)
Murakami et al. [58]	7/180	miR-18, miR-224	miR-199a*, miR-195, miR-199, miR-200a, miR-125a
Li et al. [59]	84/509	miR-106b, miR-15b, miR-18a, miR-221, miR-222, miR-224	miR-125b, miR-101
Ladeiro et al. [67]	130/250	miR-224, miR-200c, miR-203, miR-21, miR-222, miR-10b	miR-422b, miR-122a
Varnholt et al. [60]	29/80	miR-122, miR-100, miR-10a	miR-198, miR-145
Li et al. [75]	8/8	miR-17-5p, miR-18a, miR-19a, miR-20a, miR-92-1, miR-106b, miR-93, miR-25	
Pineau et al. [61]	12/215	miR-106b, miR-21, miR-210, miR-210, miR-221, miR-222, miR-224, miR-34a, miR-425, miR-519a, miR-93, miR-96	let-7c
